# Round Ligament of Uterus Leiomyoma: An Unusual Cause of Dyspareunia

**DOI:** 10.1155/2015/197842

**Published:** 2015-03-05

**Authors:** Ozer Birge, Deniz Arslan, Erdinc Kinali, Berk Bulut

**Affiliations:** ^1^Department of Obstetrics and Gynecology, Nyala Sudan-Turkish Training and Research Hospital, West Alessa District, Nyala, Sudan; ^2^Department of Urology, Nyala Sudan-Turkish Training and Research Hospital, West Alessa District, Nyala, Sudan; ^3^Department of General Surgery, Nyala Sudan-Turkish Training and Research Hospital, West Alessa District, Nyala, Sudan; ^4^Department of Obstetrics and Gynecology, Okmeydani Training and Research Hospital, Istanbul, Turkey

## Abstract

Round ligament of uterus leiomyoma is a rare, benign tumor of the vulva. Its incidence is not known exactly, and the mean age ranges from 13 to 70. Although clinical properties of benign and malignant diseases in the vulvar area are frequently similar, early diagnosis and treatment are essential. Local excision is recommended as definitive therapy. We present an 28-year-old female without any birth with a mass in anterior vaginal wall diagnosed as vulvar leiomyoma. In conclusion, a brief review of relevant literature emphasizes that leiomyomas are quite rare outside of the uterus but they might occur in any tissue or organ containing smooth muscle, spontaneously or parasitically after the spreading effect of an accident or surgical trauma. Clinicians should be alert especially for the diagnosis in a tissue with smooth muscle content.

## 1. Introduction

Tumors of the round ligament of uterus leiomyomas are benign, solid tumors of vulva which are seen rarely and usually they originate from smooth muscle fibers in ligamentum rotundum that extends to labia majora [1]. There are single case reports or case series with small numbers in the literature and the exact incidence is not known [2]. The cases are frequently encountered during fertility age and the masses enlarge throughout the pregnancy period but there are also reports of menopausal patients. Mean age varies between 13 and 70 and mean dimension of tumor has a wide range from 0,5 to 15 cm [3]. Most of the patients are asymptomatic, and in symptomatic cases with lesions located in the vulvar region, benign and malignant tumors cause similar symptoms. In this regard early diagnosis and prompt treatment are crucial. Local excision is the mode of choice for treatment [4]. We intend to discuss a patient who is admitted to the hospital with complaints of dysuria, dyspareunia, and a palpable mass in vulvar region. The pathological diagnosis is round ligament leiomyoma. The current literature is also reviewed together with the case discussion.

## 2. Case

**Figure 1:**
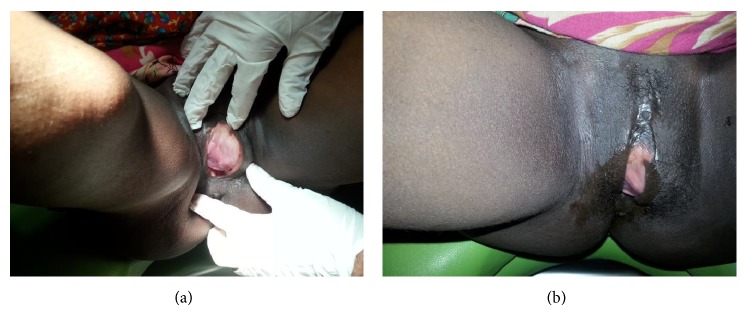
Preoperative inspection of vulvar region.

**Figure 2:**
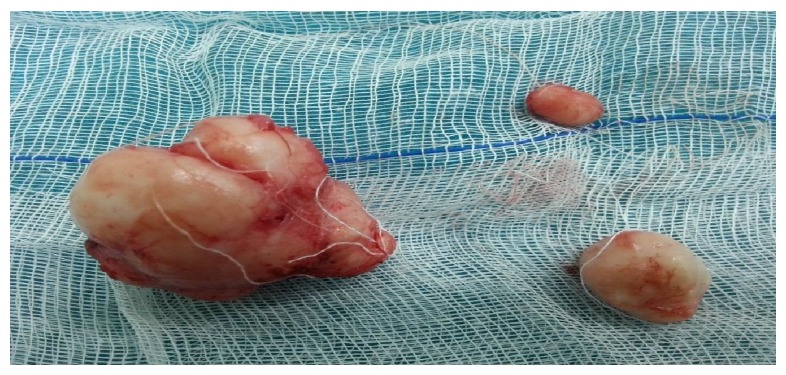
Pathological specimens.

**Figure 3:**
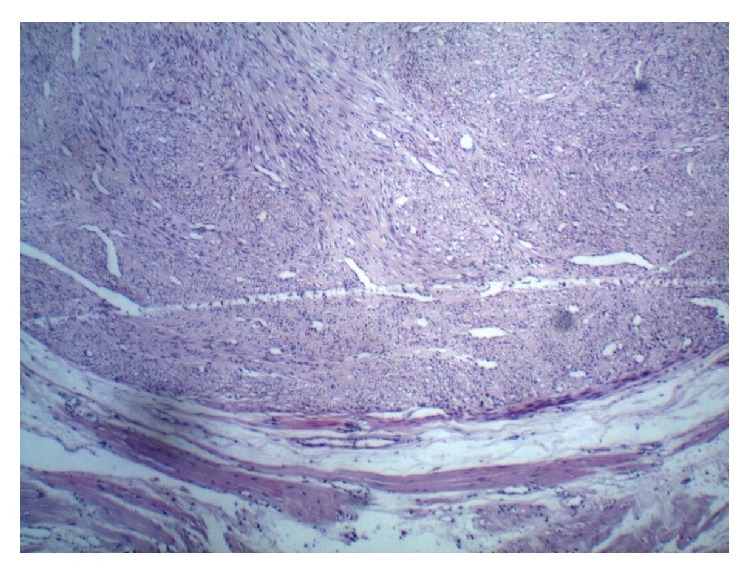
Histopathology showing whorls of smooth muscle fibres.

**Figure 4:**
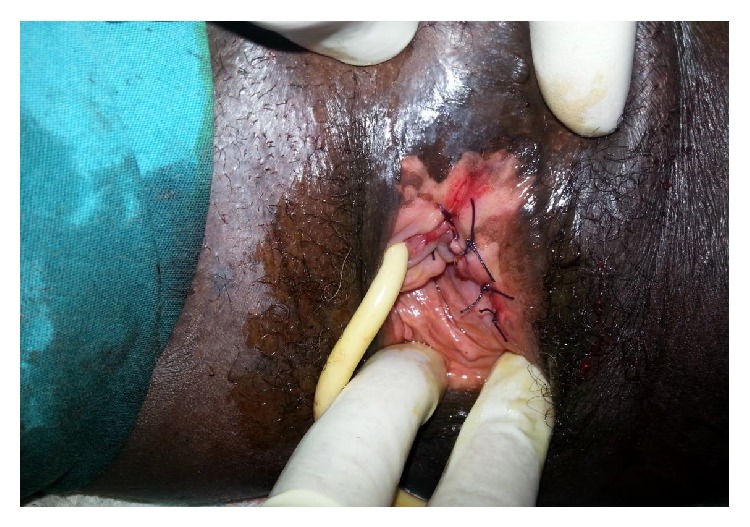
Postoperative appearance of the vulvar region.

A 28-year-old, nulligravid patient presented to our clinic with complaints of dyspareunia, dysuria, and a palpable vaginal mass. A solid, tender, irreducible mass protrudes from the anterior vaginal wall extending to left vaginal wall until the level of hymen was detected (Figures 1(a) and 1(b)). The solid mass was 5 cm in dimension and protruded completely from the anterior vaginal wall with the Valsalva maneuver. Rectal examination was normal. Transvaginal ultrasonography revealed a myomatous uterus with the largest leiomyoma being 3 × 2 cm and the ovaries seemed normal. Laboratory studies were in normal ranges for complete blood count, full biochemistry, and tumor markers. An operation was planned to remove the vaginal mass. A cystoscopy was performed preoperatively and a mass was visualized which pushed the trigone of the urinary bladder upward. In the operation, an incision of 1 cm was made on the left anterolateral wall of the vagina. After that, the 5-cm solid mass was excised totally with some hymenal tissue surrounding the tumoral structure and after operation normal anatomic imagine was seen (Figures 2 and 4). The patient was discharged on the second day with an uneventful postoperative period. Histopathocological evaluation revelaed left vulvar mass as a leiomyoma of round ligament. Round ligament of uterus leiomyoma is very rare and only few cases have been reported worldwide (Figure 3).

## 3. Discussion

Leiomyoma of the uterus is common, but leiomyoma of round ligament is rare. Fifty percent of round ligament leiomyomas present with uterine fibroids [5]. They are more frequently seen in fertility-age women, are enlarge during pregnancy, and are usually positive for estrogen and progesterone receptors histopathologically. However, there are individual cases which are diagnosed in postmenopausal period [3]. Besides, round ligament leiomyomas most commonly arise from extraperitoneal end of round ligaments, commonly on the right side [5]. The transformation of myofibromatous structure of female genital tract to leiomyoma involves somatic mutation of smooth muscles and complex interaction between sex steroids and growth factors. Estrogen is the major promoter of growth, and the role of progesterone is still unclear. It can mimic incarcerated inguinal hernia or inguinal lymphadenopathy. Surgical exploration is the treatment of choice which would differentiate between leiomyoma, inguinal lymphadenopathy, and incarcerated hernia. Smooth muscle tumors can occur in bizarre locations in the body. Rentería-Ruiz et al. reported on a Mexican woman who had a complaint of blurred vision and loss of sight in the right eye and diagnostic workup revealed a solid mass resembling an adenoma which was located in the ciliary body in the right orbita. The mass was resected and the pathology report was a mesoectodermal leiomyoma. The patient was followed by the ophthalmology department later [6]. In a comprehensive review by Omiyale done in 2014, 36 patients were identified who had a mass lesion in liver and were diagnosed with a leiomyoma after. Mean age was 43, 55.6% of the patients were females, and mean diameter of the masses was 8.7 cm. Average length of asymptomatic period was 33 months. The authors suggested that the diagnosis of primary leiomyoma was quite rare but cure could only be achieved by definitive surgery so great attention should be paid to differential diagnosis and treatment in liver masses [7]. Harish et al. published the case of a postmenopausal woman who presented with the complaints of abdominal pain and swelling in left inguinal region. The patient had undergone total hysterectomy because of uterine leiomyoma uteri 18 years before. An operation was planned to remove a soft mass of 4 × 3 cm in the left inguinal region which was thought to be a degenerated cystic mass after imaging by ultrasound and tomography. The histopathological workup revealed a benign fibroid originating from round ligament. The authors drew attention to the possibility of a newly developing leiomyoma in patients who had been operated previously for myoma uteri and the importance of a careful diagnostic workup [8]. In a case report by Midya and Dewanda, the authors presented a 35-year-old patient who presented with the complaint of a mass in the right lower quadrant which was constantly enlarging for the last 8 months. The mass had smooth contours externally and the patient had no pain. She had 3 vaginal deliveries and had undergone a laparoscopic tubal ligation 10 years before and other than these she had a nonspecific medical history. After resection of the tumor, histopathological study revealed a benign leiomyoma. In this case, tubal ligation was thought to be associated with the spreading of the smooth muscle cells into the abdominal cavity and laparoscopic operations turned out to be more frequently responsible for these kinds of consequences compared with laparotomy. Our patient had no previous surgical operations so histological resemblance between tissues might be a reason for the development of the tumor [9]. Nakamura et al. described a patient who underwent bronchoscopical resection of a 14 mm tracheal tumor after applying for difficulty in swallowing. Pathological investigation of the mass revealed a leiomyoma. The authors warned against omitting leiomyoma from the differential diagnosis of tracheal tumors even if the prevalence was very low. They also reminded that bronchoscopical resection had a higher rate of recurrence compared with open surgery [10]. Nielsen et al. proposed four criteria to identify benign and malignant lesions in their study on 25 patients: tumors ≥5 cm in greatest dimension, ≥5 mitotic figures per 10 hpf, infiltrative margins, and moderate to severe atypia. Lesions that exhibit 3 or all four criteria should be diagnosed as sarcomas and tumors manifesting two of the criteria should be categorized as benign but atypical leiomyomas and those with only one criterion are leiomyomas [11]. In our case, one of these criteria has been manifested. While sarcomas require wide excision, conservative surgery is most appropriate for leiomyomas and atypical leiomyomas with a long-term followup for recurrences. The first step is to identify whether the mass is benign or malignant and after the definite diagnosis has been made, a treatment strategy should be devised and the patient should be followed for recurrence.

## 4. Conclusion

A brief review of relevant literature emphasizes that leiomyomas are quite rare outside of the uterus but they might occur in any tissue or organ containing smooth muscle, spontaneously or parasitically after the spreading effect of an accident or surgical trauma. Clinicians should be alert especially for the diagnosis in a tissue with smooth muscle content.
